# Transcription Factor VlbZIP14 Inhibits Postharvest Grape Berry Abscission by Directly Activating *VlCOMT* and Promoting Lignin Biosynthesis

**DOI:** 10.3390/ijms25179479

**Published:** 2024-08-31

**Authors:** Peng Yu, Songqi Li, Yadan Sun, Xiangxuan Meng, Qiaofang Shi, Xiaochun Zhao, Yihe Yu

**Affiliations:** 1 College of Horticulture and Plant Protection, Henan University of Science and Technology, Luoyang 471023, China; yp864303596@163.com (P.Y.); wnlsqsy@163.com (S.L.); sunyadan@yeah.net (Y.S.); mengxiangxuanmxx@163.com (X.M.); 9906384@haust.edu.cn (Q.S.); zhaoxiaochun513@163.com (X.Z.); 2 Henan Provincial Engineering Research Center on Characteristic Berry Germplasm Innovation & Utilization, Luoyang 471023, China

**Keywords:** *VlCOMT*, VlbZIP14, berry abscission, lignin biosynthesis

## Abstract

Sulfur dioxide (SO_2_) is the most effective preservative for table grapes as it reduces the respiratory intensity of berries and inhibits mold growth. However, excessive SO_2_ causes berry abscission during storage, resulting in an economic loss postharvest. In this study, grapes were exogenously treated with SO_2_, SO_2_ + 1.5% chitosan, SO_2_ + 1.5% eugenol, and SO_2_ + eugenol-loaded chitosan nanoparticles (SN). In comparison to SO_2_ treatment, SN treatment reduced the berries’ abscission rate by 74% while maintaining the quality of the berries. Among the treatments, SN treatment most effectively inhibited berry abscission and maintained berry quality. RNA-sequencing (RNA-seq) revealed that SN treatment promoted the expression of genes related to cell wall metabolism. Among these genes, *VlCOMT* was detected as the central gene, playing a key role in mediating the effects of SN. Dual luciferase and yeast one-hybrid (Y1H) assays demonstrated that VlbZIP14 directly activated *VlCOMT* by binding to the G-box motif in the latter’s promoter, which then participated in lignin synthesis. Our results provide key insights into the molecular mechanisms underlying the SN-mediated inhibition of berry abscission and could be used to improve the commercial value of SO_2_-treated postharvest table grapes.

## 1. Introduction

Grapevine (*Vitis vinifera* L.) is a widely cultivated crop and is known for its high economic and nutritional value [1]. Grapes are rich in nutrients, such as flavone and resveratrol, which are conducive to human health, and this fruit is widely loved by consumers [2]. The total grape cultivation area worldwide reached 7 million hectares in 2022, including 840,000 hectares in China. Table grapes account for 80% of all the grapes cultivated around the world [3]. During storage and transportation, grape berries tend to fall off the fruit stalk, especially the ‘Kyoho’ (*V. vinifera* L. × *V. labrusca* L.) grape, damaging the fruit’s quality and causing severe postharvest loss [4]. With an extended storage duration, microstructural and ultrastructural changes were observed in the stem–berry junction, berry brush cells, and flower stalks of ‘Shine Muscat’ (*V. labruscana* Baily × *V. vinifera* L.) and ‘Kyoho’ grapes [5]. Therefore, it is crucial to prevent decay and postharvest berry abscission during storage to extend the shelf life of grapes.

Abscission refers to the process of separating plant tissues or organs from the plant body. Plant organ abscission is a biological phenomenon and is dependent on the genetic characteristics and developmental stages of different fruit varieties [6,7]. Abscission is an active physiological process occurring through cell wall dissolution at a predetermined location. The abscission zone is often associated with abiotic stress and aging [8]. The secretion of hydrolases, increased peroxidase activity, and the loss of calcium and pectin in the abscission layer of the cell wall can lead to the dissolution of the pectin-rich intercellular layer, weakening the strength of the cell wall [9,10].

The fruit stalks of the ‘Kyoho’ grape exhibit a highly active postharvest metabolism with rapid aging, restricting the storage of the whole fruit cluster [4]. The formation of the abscission layer, which leads to berry drop, is influenced by various physiological, biochemical, and environmental factors [11]. After harvesting the fruit cluster, the supply of growth-promoting hormones is cut off from shoot tips, leaves, and roots. This leads to hormonal imbalance and abscisic acid (ABA) accumulation [12]. Berry abscission is accompanied by changes in the activities of cell wall-degrading enzymes in the separation layer, including polygalacturonase (PG), peroxidase (POD), cellulase (Cx), and lipoxygenase (LOX) [13]. Cell wall-degrading enzymes can degrade pectin and cellulose, reducing cell adhesion [14]. ABA can enhance Cx and PG activities, accelerating the degradation of cell wall components (cellulose and pectin) [12]. On the contrary, gibberellic acid (GA_3_) and indole-3-acetic acid (IAA) suppress ABA and ethylene activities, inhibiting plant senescence and abscission [15].When the GA_3_/ABA ratio decreases to a threshold, Cx and PG activities increase, leading to the activation of abscission zone cells, degradation of cellulose and pectin, and abscission [12].

Grape berry abscission is caused by the separation of cells in the abscission zone of the pedicel [16]. The cell separation layer is gradually formed at the junction of the grape stalk and grain. At the beginning of storage, the cell separation layer at the central junction of the fruit stalk and grain is not differentiated, but the cell separation layer begins to form at the side of the fruit stalk [17]. With the extension of the storage period, both the phloem and the pulp of the stalk differentiate to produce abscission cells, but the central xylem duct does not form the abscission layer, so the weight of the fruit is supported by the xylem. If the weight of the fruit becomes intolerable, the transport tissue breaks, the fruit grain falls off, and it is serrated at the place where the fruit stalk falls off [7]. The stronger the support of the fruit stalk, the less likely the fruit will fall off [5]. In summary, to prevent ‘Kyoho’ grapes from dropping postharvest, it is crucial to strengthen the supporting ability of fruit stalks and inhibit relevant postharvest metabolism. Research indicates that ‘Kyoho’ grapes, which possess smaller fruit stem areas, are more prone to dropping compared to the ‘Red Earth’, ‘Qiu Hong’, and ‘Qiu Hei’ varieties that have larger fruit stem areas [18].

Sulfur dioxide (SO_2_) is a commonly used grape preservative, which can effectively restrain mold growth, prevent grape decay, and increase the storage time of grape berries [19]. However, studies have shown that excessive SO_2_ can damage table grapes by bleaching them and causing premature browning of the stem [20]. The decrease in stem support promotes grape berry abscission. Moreover, SO_2_ concentration during application is difficult to control. Hence, if the SO_2_ concentration is not properly regulated, it might lead to fruit abscission, reducing the commercial value of the fruit and limiting the development of the grape industry [14]. Therefore, it is crucial to devise approaches to prevent SO_2_-induced grape berry shedding. At present, chitosan is widely used in postharvest fruit preservation as it effectively prevents postharvest diseases and maintains fruit quality. As an edible coating, chitosan offers several advantages; however, the application of only chitosan for fruit preservation is associated with some drawbacks, such as postharvest fruit decay due to the inability of chitosan to inhibit specific microorganisms. Chitosan nanoparticles are more effective antibacterial agents than chitosan gel owing to the high surface area of the former and more effective interaction of charge density with the negative surface of bacterial cells. Eugenol is a natural antibacterial agent with antioxidant properties [21]. It can destroy the cell membrane of microorganisms, leading to the leakage of proteins and lipids through the cell membrane [22]. However, eugenol is highly volatile, which limits its efficiency. The encapsulation of eugenol into chitosan nanoparticles can reduce the loss of eugenol in harsh environments. However, to the best of our knowledge, the application of eugenol and eugenol-loaded chitosan nanoparticles in postharvest grape preservation has not yet been reported.

This study showed that SN treatment could maintain the storage quality of grapes postharvest and inhibit SO_2_-induced grape berry abscission. In addition, we elucidated the mechanism of action of VlbZIP14 in response to SN treatment by regulating the expression of VlCOMT. This study provided an important theoretical basis for further research on grape stalk lignin deposition and the delay of berry abscission.

## 2. Results

### 2.1. Treatment with Chitosan and Eugenol Inhibits SO_2_-Induced Berry Abscission during Storage

**Figure 1:**
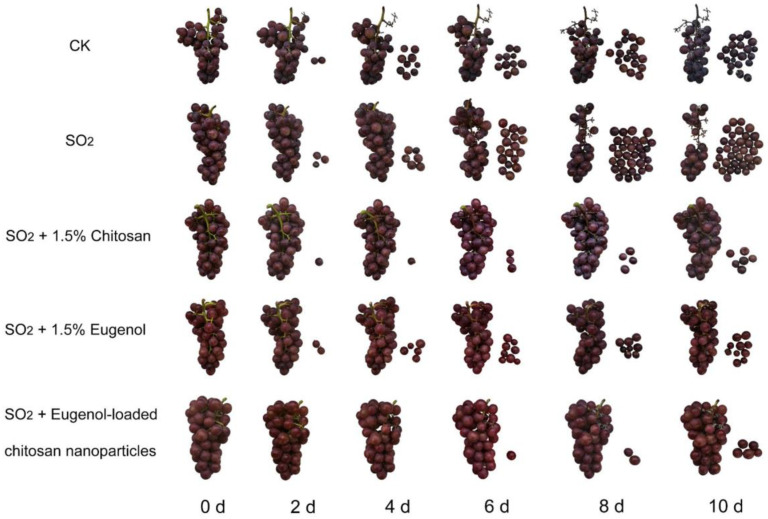
Phenotypic changes in ‘Kyoho’ grapes after exogenous treatments during storage. Treatments included the control (CK), sulfur dioxide (SO_2_), SO_2_ + 1.5% chitosan, SO_2_ + 1.5% eugenol, and SO_2_ + eugenol-loaded chitosan nanoparticles.

**Figure 2:**
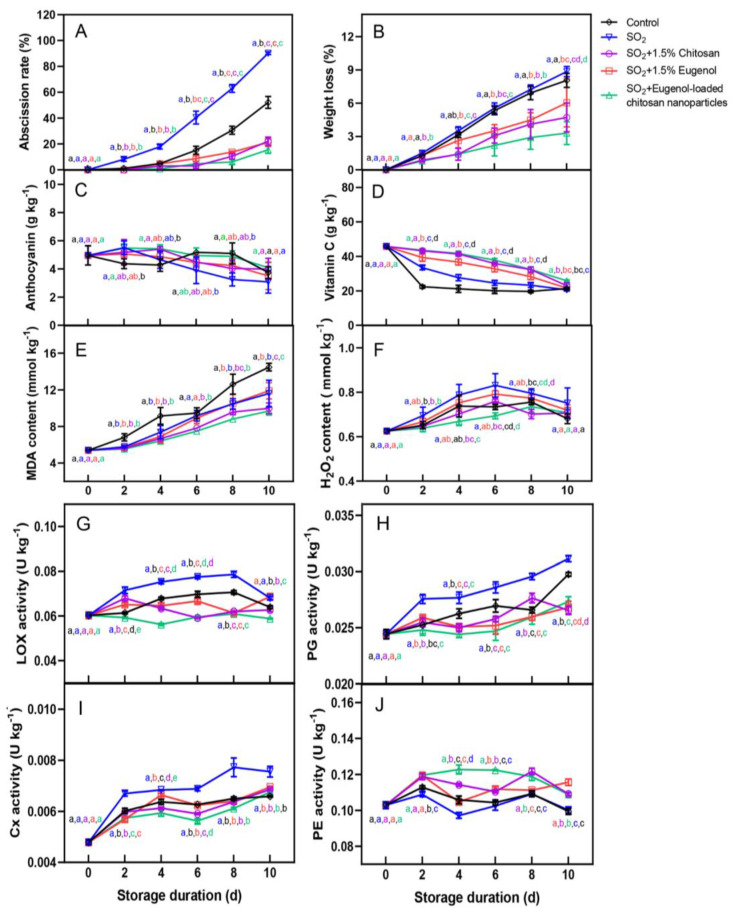
Effects of exogenous treatments on the (**A**) abscission rate, (**B**) weight loss, (**C**) anthocyanin levels, (**D**) vitamin C (VC) content, (**E**) malondialdehyde (MDA) levels, and (**F**) hydrogen peroxide (H_2_O_2_) content of table grapes. Data represent average ± SD (*n* = 3). Significant differences between means were determined using ANOVA (*p* < 0.05) and are denoted by different lowercase letters. Effects of exogenous treatments on the activities of (**G**) lipoxygenase (LOX), (**H**) polygalacturonase (PG), (**I**) cellulase (Cx), and (**J**) pectin esterase (PE) of table grapes. Data represent average ± SD (*n* = 3). Significant differences between means were determined using ANOVA (*p* < 0.05) and are denoted by different lowercase letters.

First, we observed and compared the effects of treatment with 1.5% chitosan, 1.5% eugenol, and eugenol-loaded chitosan nanoparticles on SO_2_-induced berry abscission. After 2 d of storage, we observed berry abscission in all groups except group 5, with the most prominent abscission observed for group 2, followed by the CK group. In addition, groups 3, 4, and 5 exhibited delayed berry abscission compared to the CK group and group 2 (Figure 1). We observed that SO_2_ treatment exacerbated berry abscission, with group 2 showing an abscission rate of 90% at 10 d. Compared to group 2, groups 3, 4, and 5 exhibited abscission rates of 22%, 21%, and 16%, respectively (Figure 2A). These results showed that chitosan, eugenol, and SN treatments inhibited SO_2_-induced fruit abscission, with the SN treatment exerting the most prominent inhibitory effect on berry abscission.

### 2.2. Treatment with Chitosan and Eugenol Maintains the Quality of Table Grapes during Storage

The fruit weight loss gradually increased with increasing storage time. CK and group 2 grapes exhibited the most prominent weight loss, with weight loss rates reaching 8% and 9% at 10 d, respectively. However, groups 3, 4, and 5 exhibited weight loss rates of 5%, 6%, and 3%, respectively (Figure 2B). During storage, groups 2–5 exhibited comparable decreases in the anthocyanin levels of grapes (Figure 2C). Furthermore, all groups exhibited a decrease in vitamin C (VC) levels with increasing storage time, with more a prominent decrease observed for the CK group, followed by group 2 (Figure 2D). Groups 3–5 exhibited a delayed decline in VC content.

### 2.3. Treatment with Chitosan and Eugenol Reduces the Lipid Peroxidation of Table Grapes during Storage

The accumulation of H_2_O_2_ and MDA in fruit can accelerate plant senescence. We observed a gradual increase in the MDA levels in each group with increasing storage time (Figure 2E). Among the groups, the CK group exhibited the most prominent MDA accumulation, followed by group 2. Compared to group 2, groups 3–5 exhibited lower MDA accumulation during storage. However, the inhibitory effect of the 1.5% eugenol on MDA accumulation was not significant. Furthermore, all the groups exhibited an increase in H_2_O_2_ content, followed by a decrease. H_2_O_2_ levels in group 2 peaked at day 6 of storage. Moreover, groups 3–5 exhibited lower H_2_O_2_ levels than group 2 at all time points (Figure 2F).

### 2.4. Treatment with Chitosan and Eugenol Inhibits the Activity of Cell Wall-Degrading Enzymes of Table Grapes

The activities of cell wall-degrading enzymes are closely related to fruit abscission. Thus, we determined LOX, PG, Cx, and PE activities in ‘Kyoho’ grapes during storage. We observed that all groups generally showed a gradual upward trend in LOX, PG, and Cx activities (Figure 2G–I). However, group 2 exhibited higher LOX, PG, and Cx activities than the CK group. Compared to group 2, groups 3–5 exhibited marked inhibition of the increase in PG activity. Similarly, groups 3–5 exhibited lower LOX and Cx activities than group 2 at all time points. These results indicated that chitosan, eugenol, and SN treatments inhibited the activities of cell wall-degrading enzymes in grapes.

### 2.5. Transcriptome Analysis of SO_2_-Induced Abscission of Berries Treated with Eugenol-Loaded Chitosan Nanoparticles

Based on the physiological data and observed phenotypes, the SN treatment most prominently inhibited berry abscission and maintained fruit quality. To determine the mechanism underlying the inhibition of fruit abscission post-SN treatment, transcriptome analysis was conducted on samples from groups 1, 2, and 5 collected at 2, 4, and 6 d post-treatment. The total mRNA was sequenced through the Illumina NovaSeq 6000 platform, and the sequencing comparison rates between the clean reads of the samples and the specified reference genome ranged between 82.48% and 86.34%.

**Figure 3:**
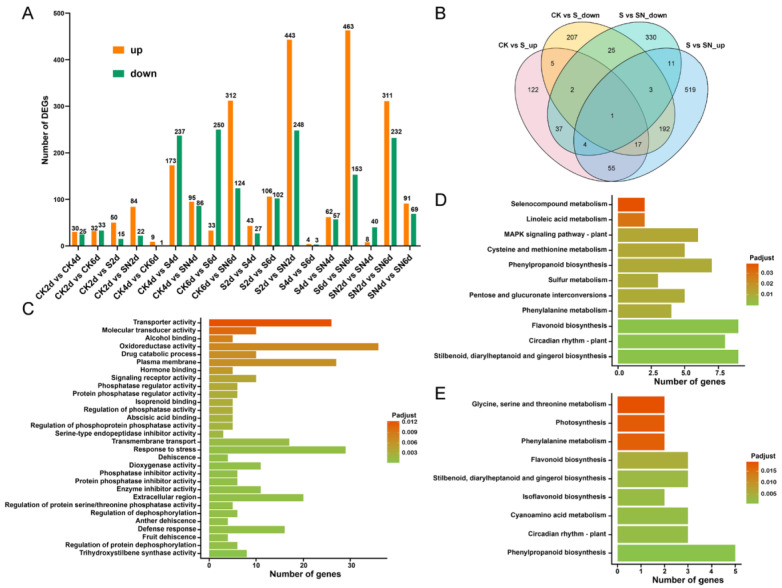
The number of differentially expressed genes (DEGs) at different storage periods. (**A**) Comparison of the number of upregulated and downregulated DEGs under different treatments. The *X*-axis indicates the storage period of berries, and the *Y*-axis shows the number of DEGs. S: Sulfur dioxide (SO_2_) treatment; SN: SO_2_ + eugenol-loaded chitosan nanoparticles edible coating treatment. (**B**) Venn diagram showing the number of DEGs. (**C**,**D**) A total of 213 genes were enriched as determined by GO enrichment and KEGG pathway analysis. The *X*-axis indicates the number of enriched DEGs, and the *Y*-axis shows the GO terms. (**E**) A total of 44 DEGs were examined via the KEGG pathway analysis. The *X*-axis indicates the number of enriched genes, and the *Y*-axis indicates the KEGG pathways.

According to the criteria of p-adjust < 0.05 & |log_2_FC| ≥ 1, we detected 1949 DEGs in the three grape groups at the three time points (Figure 3A and Figure S3). Next, we determined the genes that were co-expressed during SO_2_-mediated induction and SN-mediated inhibition of the berry abscission pathway. We identified 213 overlapping genes by comparing the genes that were downregulated after SO_2_ treatment with those that were upregulated after SN treatment and 44 overlapping genes by comparing the genes that were upregulated after SO_2_ treatment with those that were downregulated after SN treatment (Figure 3B). GO and KEGG pathway enrichment analyses were performed on the 213 co-expressed DEGs. The GO terms primarily included trihydroxystilbene synthase activity, fruit dehiscence, defense response, response to stress, abscisic acid binding, polygalacturonase activity, cinnamic acid metabolism, phenylalanine ammonia-lyase activity, and abscisic acid-activated signaling pathway (Figure 3C). A total of 11 pathways were enriched, including stilbenoid, diarylheptanoid, and gingerol biosynthesis; flavonoid biosynthesis; phenylalanine metabolism; and cysteine and methionine metabolism (Figure 3D). Next, the 44 DEGs were examined via the KEGG analysis and were enriched in nine pathways, including phenylpropane biosynthesis; isoflavone biosynthesis; stilbene, diarylheptane, and gingerol biosynthesis; flavonoid biosynthesis; and phenylalanine metabolism and photosynthesis (Figure 3E).

### 2.6. SN Treatment-Related Modules Screened by WGCNA

**Figure 4:**
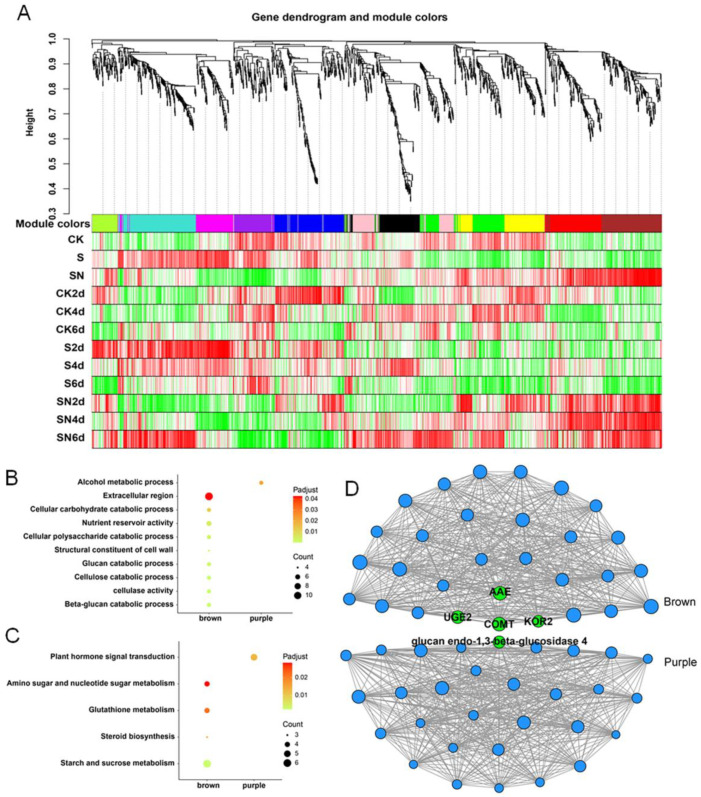
Weighted gene co-expression network analysis (WGCNA) of DEGs at different storage periods. (**A**) The correlations between genes in different modules and under different treatments. The upper part shows the hierarchical clustering tree of genes, the middle part shows the modules, and the lower part shows the heatmap of the correlations between genes and processes in each module. Each row represents a phenotype, and each column represents a gene in the module. Color indicates the magnitude of the correlation, with red corresponding to a positive correlation and green corresponding to a negative correlation. S: Sulfur dioxide (SO_2_) treatment; SN: SO_2_ + eugenol-loaded chitosan nanoparticles edible coating treatment. (**B**,**C**) GO enrichment and KEGG pathway analysis of the modules. (**D**) Visualization of the correlation between the brown and purple module genes. Each node represents a gene. The size of the nodes increases as the characteristic gene connectivity (kME) increases.

WGCNA analysis was used to assess the regulatory network of the 1949 DEGs produced by CK, SO_2_, and SN treatment. Genes with similar expression patterns were classified into one module. Finally, 12 gene modules were constructed (Figure 4A). Through the screening criteria of Pearson correlation coefficient (r) > 0.8 and *p*-value (*p*) < 0.05, the characteristic modules strongly related to different treatments or storage periods were determined. The brown module significantly positively and negatively correlated with the SN (r > 0.8) and SO_2_ treatments (r < −0.6), respectively. The purple module negatively correlated with the SN treatment (r < −0.8, Figure 4B). To investigate the molecular mechanism of genes in characteristic modules, GO and KEGG enrichment analyses were carried out on the brown and purple modules to determine the role of genes in specific biological processes and metabolic pathways. Based on the criteria of p-adjust < 0.05, nine GO terms were enriched in the brown module, including cellulose catabolic process, *β*-glucan catabolic process, cellulase activity, glucan catabolic process, structural constituent of cell wall, and cellular polysaccharide catabolic process, which are related to cell wall metabolism. The enriched KEGG pathways included starch and sucrose metabolism, steroid biosynthesis, glutathione metabolism, and amino sugar and nucleotide sugar metabolism. A single GO term and KEGG pathway were enriched in the purple module (Figure 4C,D).

To investigate the key regulatory genes in the WGCNA modules, a regulatory network diagram was constructed to analyze the relationships between the genes in the brown and purple modules. Genes with high characteristic gene connectivity (kME) were shown in the brown and purple modules. Three genes were related to cell wall metabolism, including caffeic acid-O-methyltransferase (*COMT*, Vitvi02g00263), endo-1,4-*β*-glucanase korrigan (*KOR2*, Vitvi00g01392), and UDP-glucose 4-epimerase GEPI48 (*UGE2*, Vitvi02g00143) (Figure 4D and Figure S4).

### 2.7. COMT Promotes the Synthesis of Lignin and Inhibits Berry Abscission

**Figure 5:**
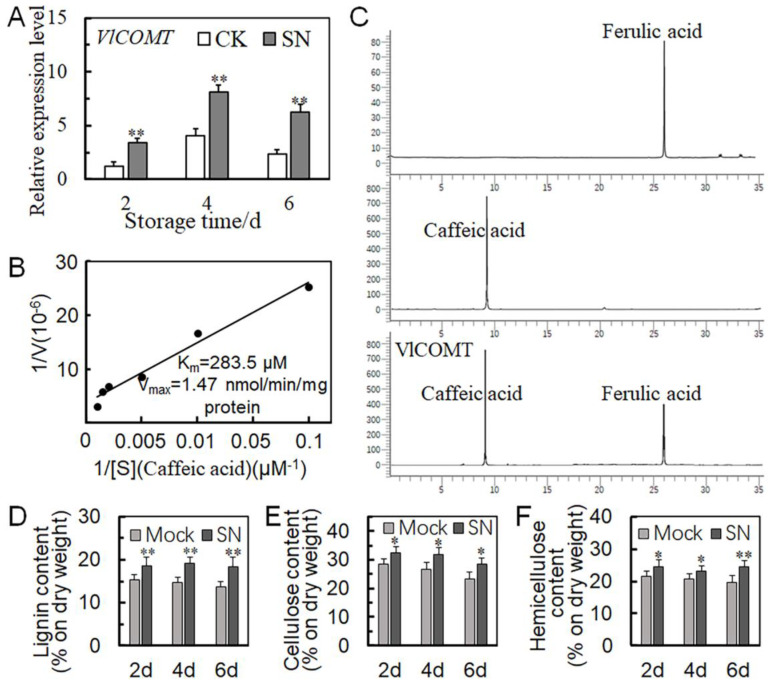
Effects of *VlCOMT* expression levels on lignin synthesis in grapes. (**A**) Quantitative real-time polymerase chain reaction (qRT-PCR) results of *VlCOMT* at three different storage periods. (**B**) Determination of caffeic acid-O-methyltransferase activity. The x-coordinate is the substrate concentration, the y-coordinate is the enzymatic reaction rate, and Vmax is the maximum reaction rate. The Michaelis constant (Km) refers to the concentration of substrate (S) at half the maximum velocity (Vmax) of the enzymatic reaction. (**C**) Determination of caffeic acid catalyzed by the VlCOMT enzyme using high-performance liquid chromatography (HPLC). The two upper graphs represent controls. The peaks are the wavelengths detected by HPLC for caffeic acid and ferulic acid. The third graph shows the presence of caffeic acid and ferulic acid after adding caffeic acid-O-methyltransferase. (**D**–**F**) Determination of lignin, cellulose, and hemicellulose contents. Each determination was repeated thrice and averaged. Mock: Blank control, SN: Sulfur dioxide (SO_2_) + eugenol-loaded chitosan nanoparticles. Each column represents the mean ± SD of three biological replicates. * *p* < 0.05, and ** *p* < 0.01 between SN treatment and mock or CK (Student’s *t*-test).

The co-expression network showed that *VlCOMT* was the key gene in the center of the network. We speculate that *VlCOMT* might play an important role in regulating cell wall metabolism. To investigate whether *VlCOMT* was differentially expressed by SN treatment and is involved in cell wall metabolism, *VlCOMT* mRNA abundance was analyzed by qRT-PCR. The results indicated that compared with the control group, *VlCOMT* was upregulated in the SN treatment group (Figure 5A). Previous studies have shown that COMT is a key enzyme in the lignin synthesis pathway and can catalyze caffeic acid to form ferulic acid. Ferulic acid and caffeic acid are the precursors of lignin synthesis [23]. To investigate whether *VlCOMT* is involved in lignin synthesis, we analyzed the activity of caffeic acid-O-methyltransferase and detected whether VlCOMT can catalyze caffeic acid to ferulic acid by using HPLC. Our results showed that the activity of methyltransferase increased with increasing caffeic acid levels, indicating that caffeic acid-O-methyltransferase catalyzes caffeic acid (Figure 5B). HPLC showed that ferulic acid and caffeic acid were detected after the addition of caffeic acid-O-methyltransferase, indicating that caffeic acid-O-methyltransferase catalyzed the formation of ferulic acid, participating in lignin synthesis (Figure 5C). These findings indicated that SN treatment might induce *VlCOMT* upregulation, thus promoting lignin formation and enhancing the support of fruit stalks. Therefore, we determined lignin, cellulose, and hemicellulose levels in the dry weight of the SN treatment and CK groups. The dry weight measurements showed higher lignin, cellulose, and hemicellulose levels in the SN treatment group than in the CK group (Figure 5D–F).

### 2.8. VlCOMT Overexpression Promotes Lignin Synthesis

**Figure 6:**
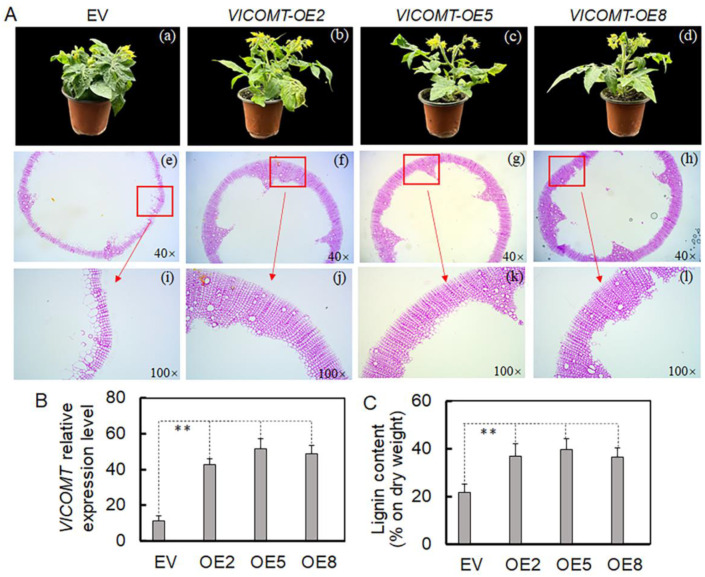
(**A**) (**a**–**d**) The phenotype of transformed with empty vector (EV) and *VlCOMT*-overexpressing tomato plants. (**e**–**l**) Detection of the autofluorescence of lignin under ultraviolet light. Both wild-type and (*VlCOMT*-overexpression) *VlCOMT*-*OE* lines were cultured twice, and sections were taken from three independent plants for paraffin embedding. The results of the transgenic lines are similar. (**B**) *VlCOMT* expression level in the OE lines. (**C**) The lignin content in the OE lines. Each column represents the mean ± SD of three biological replicates. ** *p* < 0.01 between transgenic plants and CK plants (Student’s *t*-test).

To further study the function of *VlCOMT* in lignin synthesis, Agrobacterium-mediated transformation was used to transform the cotyledon explants of *Micro*-*Tom* tomatoes. However, no significant differences were observed in their appearance after transformation. Further, the transverse sections of the tomato’s main stem were embedded in paraffin and sliced. Detection of the autofluorescence characteristics of lignin under a laser scanning confocal microscope (LSCM) revealed that the autofluorescence of lignin in *VlCOMT-OE* lines was significantly enhanced, and the secondary cell walls of vessels and interfascicular fibers were significantly thickened. Further analysis showed a significant increase in the lignin content of *VlCOMT-OE* lines, indicating that *VlCOMT* can promote lignin synthesis in tomato stems (Figure 6).

### 2.9. VlbZIP14 Specifically Activates the VlCOMT Promoter

**Figure 7:**
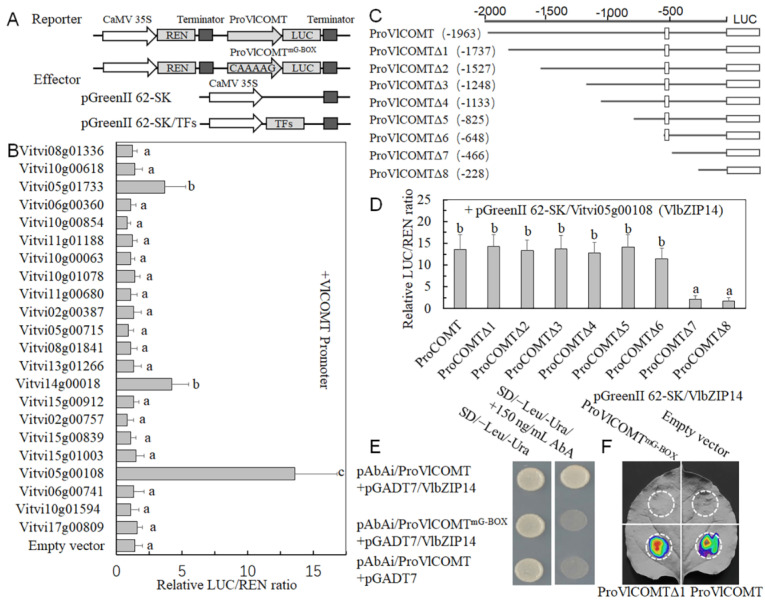
*VlbZIP14* directly binds to the G-box motif on the *VlCOMT* promoter to activate its expression. (**A**) Construct vector for dual luciferase assays. (**B**) The TF_S_ effectors containing the *VlCOMT* promoter reporter were infiltrated into the *N. benthamiana* leaves. Compared with the empty vector, the LUC/REN ratio of the VlbZIP14 effect vector was significantly increased, indicating that *VlbZIP14* enhanced *VlCOMT* promoter activity. (**C**) Schematic diagram of a vector with a partially missing *VlCOMT* promoter. (**D**) Fragments with partially deleted *VlCOMT* promoter and effector *VlbZIP14* were infiltrated into *N. benthamiana* leaves. Only the *VlCOMT* promoter fragment with a G-box could significantly improve the LUC/REN ratio of the *VlbZIP14* effector vector, and the LUC/REN ratio of the *VlCOMT* fragment without a G-box was very low. (**E**) Yeast one-hybrid (Y1H) assay of *VlbZIP14* binding to *VlCOMT* promoter fragments. The vectors containing *VlCOMT* fragments with a normal G-box and a G-box with the C/A/A/A/G mutation and the vector containing *VlbZIP14* were introduced into yeast strain Y187. The bait vectors ProVlCOMT + pGADT7 were transformed into Y1H as a negative control. (**F**) The binding of *VlbZIP14* to the *VlCOMT* promoter was determined by dual luciferase assay. The color scale shows the range of luminescence intensity. Each column represents the mean ± SD of three biological replicates. Different letters indicate significant differences (one-way ANOVA with Duncan’s multiple-range test, *p* < 0.05).

In our previous study, we selected 22 transcription factors from the transcriptome data that may play a key regulatory role in the inhibition of berry abscission by SN treatment. In the present study, a dual luciferase assay was performed to further confirm whether these 22 transcription factors specifically regulate *VlCOMT*. Our results showed that only VlbZIP14 (Vitvi05g00108) exhibited responsive activation activity for the *VlCOMT* promoter (Figure 7A,B).

The dual luciferase assay was also used to determine the cis-acting element of *VlbZIP14* that binds to the *VlCOMT* promoter. Eight *VlCOMT* promoter fragments were analyzed, and *VlbZIP14* only bound to the fragments containing the G-box motif (Figure 7C,D). Next, a Y1H assay was performed to confirm whether *VlbZIP14* specifically bound to the G-box motif on the *VlCOMT* promoter. Our results showed that the yeast cells co-expressed with the prey and bait plasmids exhibited normal growth on the selective medium. However, yeast cells with a mutated G-box motif could not grow on the selective medium (Figure 7E). Consistently, we used a dual luciferase assay to measure luminescence. Similar to the results of Y1H, the DNA fragments containing the normal and mutated G-box motif sequences were separately fused with the firefly LUC gene to construct promoter-LUC reporter plasmids. Together with the VlbZIP14 effector construct, the promoter-LUC reporter plasmids were expressed transiently in N. *benthamiana* leaves via *Agrobacterium* GV3101 (pSoup)-mediated transfection. The infiltrated leaves containing the normal G-box motif showed stronger LUC activity, while those containing the mutated motif showed no LUC activity. These results indicated that the G-box motif is essential for *VlCOMT* transcription and is regulated by *VlbZIP14* (Figure 7F).

**Figure 8:**
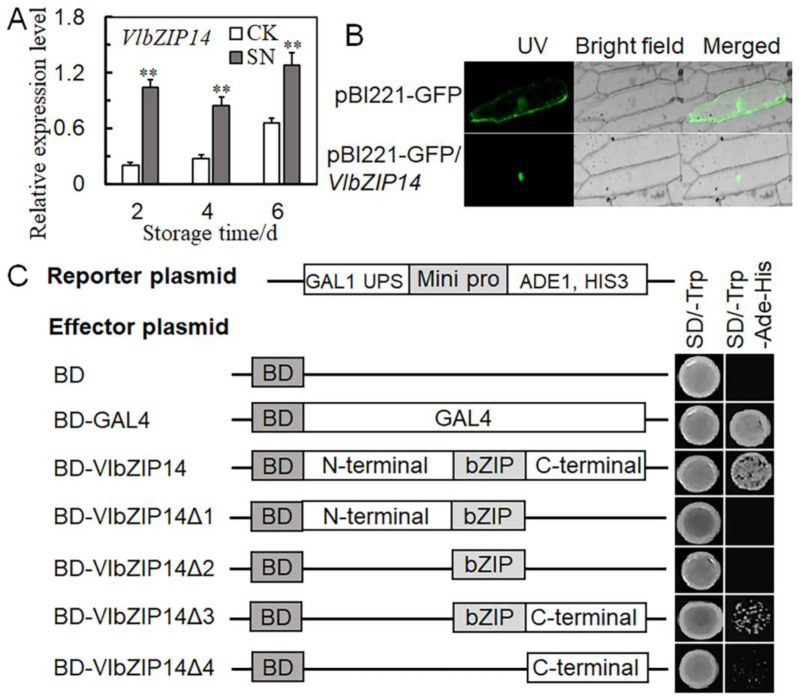
Subcellular localization of VlbZIP14 and transactivation analysis of *VlbZIP14* in yeast. (**A**) Quantitative real-time polymerase chain reaction (qRT-PCR) results of *VlZIP14* at three different storage periods. (**B**) Subcellular localization images of the control plasmid pBI221-GFP (top) and pBI221-GFP/VlbZIP14 (bottom) constructs. The scale bar used was 200 μm. (**C**) BD-VlbZIP14 was truncated to obtain BD-VlbZIP14Δ1, BD-VlbZIP14Δ2, BD-VlbZIP14Δ3, and BD-VlbZIP14Δ4 as effector plasmids. SD-Trp-His-Ade was used as a selective medium. Each column represents the mean ± SD of three biological replicates. ** *p* < 0.01 between SN treatment and CK (Student’s *t*-test).

The qRT-PCR results showed that *VlbZIP14* was significantly upregulated post-SN treatment (Figure 8A). To determine the subcellular localization of VlbZIP14, we transiently expressed a VlbZIP14-GFP fusion protein in the onion epidermis. The VlbZIP14-GFP protein was detected exclusively in cell nuclei, suggesting that VlbZIP14 is a nuclear protein (Figure 8B). Next, the transcriptional activity of VlbZIP14 was verified in yeast (AH109). Full-length VlbZIP14 and its N- (BD-VlbZIP14Δ1) and C-terminal domains (BD-VlbZIP14Δ3) were fused to the C-terminus of the GAL4 DNA binding domain (BD-GAL4). Yeast AH109 cells expressing the empty vector, BD-GAL4, BD-VlbZIP14Δ1, BD-VlbZIP14Δ2, BD-VlbZIP14Δ3, and BD-VlbZIP14Δ4 were generated. Yeast cells transformed with BD-GAL4, BD-VlbZIP14, and BD-VlbZIP14Δ3 grew well on SD/-Trp-Ade-His medium, whereas the cells containing the empty vector, BD-VlbZIP14Δ1, BD-VlbZIP14Δ2, and BD-VlbZIP14Δ4 did not. These results showed that VlbZIP14 is a transcriptional activator and that the C-terminal part of the protein is required for its transcriptional activation activity (Figure 8C).

## 3. Discussion

The berry abscission caused by excessive SO_2_ during storage limits the economic value of grapes. Edible coatings effectively prevent fresh fruit from contacting the air and reduce respiration, enhancing fruit preservation. These coatings are non-toxic, non-polluting, and low-cost. Chitosan is a biodegradable polymer widely used in such edible coatings [24]. For instance, an active biodegradable coating based on chitosan inhibits fruit decay and the activity of cell wall-degrading enzymes, promoting antibacterial activity and prolonging the shelf life of fresh strawberries [25]. Chitosan-based coating can also reduce fruit weight loss, delay the change in color and titratable acid level, and extend the shelf life of strawberries and red raspberries during cold storage [26]. The combined coating of 3% CaCl_2_ and nano-chitosan positively impacts the physical properties of strawberry fruit, with a significant fruit preservation effect [27]. Previous studies have reported that treatment with 1.5% chitosan can effectively inhibit SO_2_-induced grape abscission. However, whether other types of exogenous treatments exert a similar effect remains unclear. In this study, we compared the efficacies of 1.5% chitosan, 1.5% eugenol, and eugenol-loaded chitosan nanoparticles in inhibiting grape abscission (Figure 1 and Figure 2A). These results showed that eugenol-loaded chitosan nanoparticles exerted the most prominent inhibitory effects on the abscission of ‘Kyoho’ grapes, which indicated that eugenol-loaded chitosan nanoparticles have similar effects as chitosan. This finding might be attributed to the ability of the nanoparticles to form an edible coating on the surface of the berry and fruit stalk, providing protection from the deleterious effects of the SO_2_ gas.

The maintenance of fruit quality during fruit storage is important, but membrane lipid peroxidation destroys the integrity of the cell membrane, causes fruit aging, and reduces fruit quality [28]. H_2_O_2_ and MDA are the main oxidizing substances in cells, and their accumulation can accelerate fruit senescence [29]. These results showed that the three exogenous treatments could prevent cell oxidation, delay fruit senescence and deterioration, and help maintain grape quality (Figure 2E,F). We also determined the VC levels in grapes. VC is one of the nutritional components of fruit and also an important antioxidant for removing reactive oxygen species in fruit, delaying senescence [30]. Among all the groups in the present study, the SN treatment group exhibited the highest VC content in grape berries (Figure 2D).

The plant cell wall is composed of cellulose, hemicellulose, pectin, lignin, and other phenolic compounds [31]. With increasing storage time, the fruit ripens continuously, altering cell wall structure, decreasing intercellular adhesion, and leading to pectin degradation and fruit softening. Cell wall metabolism-related genes play a key role in this process, including PG, xylogalacturonan, β-galactosidase (β-Gal), putative UDP-glucose-4-epimerase, Cx, and α-arabinofuranosidase. The genes encoding pectin-metabolizing enzymes, such as those encoding pectin methylesterase and β-Gal, have been confirmed to be involved in abscission. Furthermore, previous studies [32] have shown that berry abscission is related to the increased activity of hydrolases in the abscission zone, especially Cx and PG. These enzymes may work together or synergistically to delay the degradation of the middle lamellae and primary cell walls in the fruit abscission zone. This finding was in line with the results of previous studies [33]. The activities of the cell wall-degrading enzymes LOX, PG, and Cx positively correlated with the shedding rate of postharvest grape berries [34]. The three exogenous treatments decreased the activities of the cell wall-degrading enzymes and the abscission rate of berries (Figure 2G–I).

To further explore the molecular mechanism underlying the inhibition of berry abscission, SN treatment was applied to grapes for transcriptome analysis. Samples were obtained at 2, 4, and 6 d post-treatment because this time period covered the early stage of the inhibition of berry abscission by SN treatment. Transcriptome analysis showed that SN treatment significantly impacted the expression of genes related to cell wall metabolism (Figure 3). These genes were upregulated in the SN treatment group compared with the CK group. The 1949 genes were further analyzed via DEG, TCseq, and WGCNA analyses to determine the key pathways and candidate factors related to the inhibition of berry abscission (Figure 4). The GO analysis of these genes showed that the GO terms were primarily related to cell wall metabolism, such as polygalacturonase activity, *β*-glucan catabolic process, cellulase activity, cellulose catabolic process, galactose metabolic process, UDP-glycosyltransferase activity, and structural constituent of cell wall. In KEGG analysis, the enriched pathways included phenylpropane biosynthesis, flavonoid biosynthesis, and isoflavone biosynthesis pathways. The phenylpropane pathway involves the synthesis of secondary metabolites, such as lignin, flavonoids, and phenolic volatiles [35]. This finding suggested that the SN treatment might govern berry abscission by regulating the cell wall metabolic pathway. In addition, recent studies have shown that the ethylene-responsive factor LcERF2 regulates litchi abscission by regulating the expression of cell wall metabolism genes [9]. Consistent with this finding, our results showed that cell wall metabolism-related genes might play a key role in inhibiting SO_2_-induced berry abscission. In one study, RNA-seq analysis revealed an upregulation of cell wall remodeling-related gene expression during the abscission process of sweet cherries, highlighting their crucial role in regulating this process. These findings are consistent with our results [36]. Therefore, we focused on the genes related to cell wall metabolism.

Lignin, cellulose, and hemicellulose are the main components of the plant skeleton. Lignin is primarily involved in compressive resistance. Hemicellulose is located between lignin and cellulose fibers, acting as a connection and forming a very strong cellulose–hemicellulose–lignin network structure. In the present study (Figure 5D–F), the SN treatment group exhibited higher levels of these cell wall components than the CK group, indicating that SN treatment delayed the degradation of lignin, cellulose, and hemicellulose and maintained the supporting force of grape stalks to a certain extent.

Transcriptome analysis revealed three genes associated with cell wall synthesis with kME > 0.8, including *COMT*, *KOR2*, and *UGE2*. Among these, *COMT* was located at the center of the network diagram (Figure 4D). Furthermore, the SN treatment group exhibited higher *VlCOMT* expression than the CK group (Figure 5A). Moreover, *COMT* is a key enzyme in the lignin-specific pathway, catalyzing the transformation of caffeic acid to ferulic acid. *VlCOMT* overexpression in tomato and its role in the synthesis of S-lignin can upregulate lignin synthesis (Figure 5B and Figure 6). Lignin is a phenolic compound deposited in the lignified cell wall, which exists in the secondary wall in the form of high-strength cross-links, thus enhancing the strength of the cell wall and providing mechanical support for whole plant organs and tissues while forming a conduit to transport water and minerals [34,37]. Some studies have shown that elevated lignin content in plant tissues can enhance the pull and support of plant organs. For instance, *PICOMT1* overexpression in peony can promote lignin accumulation and increase the thickness of secondary cells in the xylem, thus enhancing the stem strength of the plant [38]. In rapeseed, the formation of lignified adhesion structures reduces fruit cracking and falling grain [39]. In rice, lignin deposition and abscission in the separation layer reduce the risk of grain drop [40].

**Figure 9:**
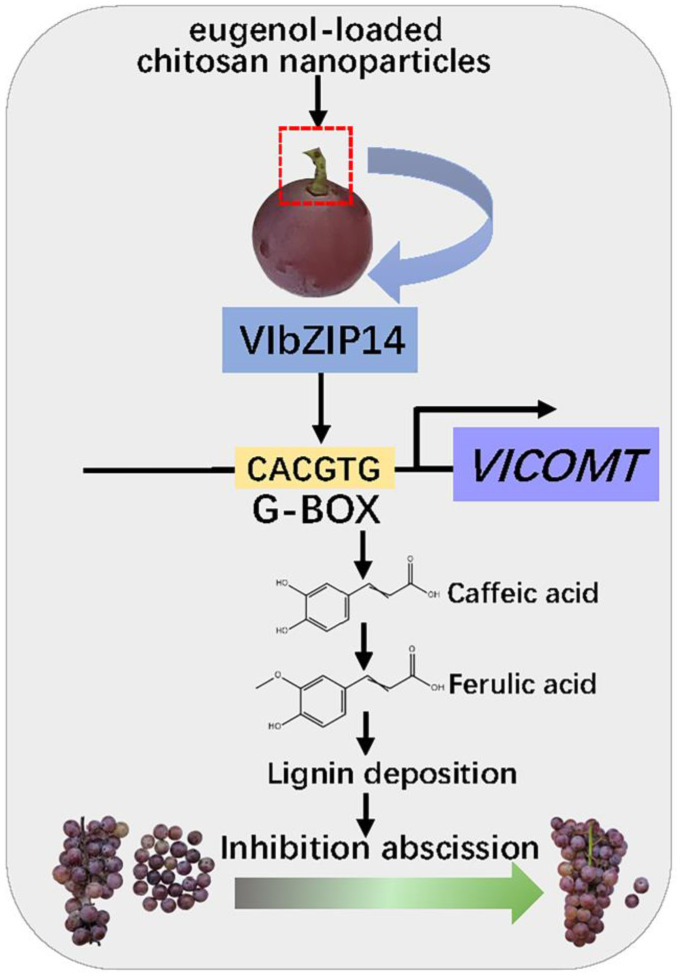
Model depicting *VlCOMT*-mediated regulation of lignin synthesis under SN treatment in ‘Kyoho’ grapes. Under SN treatment, the model proposes that the grape transcription factor *VlbZIP14* activates *VlCOMT* transcription by binding to the G-box in the latter’s promoter, increasing the lignin content of the grape stalk, enhancing the support of the grape stalk, and delaying the abscission of the berries.

Previous studies have indicated that bZIP transcription factors regulate several growth and developmental processes and abiotic stress responses [41]. G-box has a high affinity for the bZIP binding element [42]. The bZIP transcription factor *OsABI5* in rice binds to the G-box element to transactivate reporter gene expression. The N-terminus of *OsABI5* is necessary for its activation activity [43]. In the present study, we found a G-box motif within the *VlCOMT* promoter sequence. We used in vitro experiments, such as Y1H and dual luciferase assays, to determine whether *VlbZIP14* can bind to the G-box motif in the *VlCOMT* promoter and observed that the N-terminal domain is essential for the activation activity of *VlbZIP14* (Figure 7C–F), which was consistent with previous studies. Similar to the expression trend of *VlCOMT*, the SN treatment group exhibited a higher *VlbZIP14* expression than the CK group (Figure 8A). We speculated that *VlbZIP14* might promote lignin synthesis by inducing *VlCOMT*, thus enhancing the support of the grape stalk and delaying grape berry abscission (Figure 9).

## 4. Methods

### 4.1. Plant Materials and Exogenous Treatment

‘Kyoho’ grapes (*V. vinifera* L. × *V. labrusca* L.) were obtained from a vineyard in Yanshi, Luoyang, China. The annual average temperature where they were grown is 14.2 °C, the minimum temperature is −8.1 °C, and the average frost-free period is 211 days. The annual average rainfall is 500–600 mm, and the total annual sunshine hours are 2248.3 h. Grapes of similar maturity and uniform color and size were selected and transferred to the laboratory immediately after harvesting. The collected grapes were divided into five groups, with a total of six clusters in each group. From each group, three clusters were used to assess the phenotype, abscission rate, and weight loss rate of the grapes, and the remaining three clusters were used to determine the physiological indexes. To eliminate the impact of potentially confounding factors, grape clusters (weighing approximately 500 g) of similar size and color were soaked in 0.5% (*w*/*v*) sodium hypochlorite (NaClO) solution for 2 min for disinfection. After drying the grape surface, the groups were treated as follows: Group 1: The grapes were soaked in distilled water for 2 min to be used as controls (CK). Group 2: The grapes were treated with the CT-2 grape preservative (the main ingredient is SO_2_) to be used as the SO_2_ treatment group. Groups 3–5: The grapes were treated with solutions containing either 1.5% chitosan (group 3), 1.5% eugenol (group 4), or eugenol-loaded chitosan nanoparticles (group 5) for 2 min. The clusters were then air dried and laid on plastic trays. Then, they were placed into polyethylene (PE) bags containing the CT-2 grape preservative. 

Finally, all the treated grapes were wrapped in aluminum foil and stored at 20 °C. The CT2-grape preservative was used for SO_2_ treatment. The main component of the preservative was SO_2_. Four preservative tablets were used per kg of grape clusters. Briefly, a 1 mm needle was used to form a hole in the CT-2 packaging bag. The SO_2_ gas was then continuously released into the PE bag to form an excessive SO_2_ gas environment. The grapes were then kept in the PE bag for SO_2_ exposure.

The stored grapes were collected at 2, 4, and 6 days post-treatment. The samples were then placed in liquid nitrogen and stored in a −80 °C refrigerator. For sampling, 25% of the grapes were cut and used (from the end connecting to the fruit stem) for physiological assessment and RNA-seq analysis. Three replicates were used for each treatment, with 15 samples per replicate.

Chitosan with a deacetylation degree greater than 95% (Hefei Bomei Biotechnology Co., Ltd., Hefei, China) was dissolved in acetic acid solution (1% *v*/*v*) and stirred for about 12 h to prepare 1.5% (*w*/*v*) chitosan solution. Eugenol (purity 99%, Cool Chemical Technology Co., Ltd., Beijing, China) was gradually dropped into acetic acid solution (1% *v*/*v*) and stirred evenly to prepare 1.5% (*w*/*v*) eugenol solution. Eugenol-loaded chitosan nanoparticles were prepared via a two-step method (chitosan:eugenol = 1:1) as previously described by Woranuch et al. [44].

### 4.2. Abscission Rate and Weight Loss

The abscission rate was determined using the following formula: (weight of dropped berries/total weight of the cluster) × 100%. The weight loss was determined using the following formula: [(initial weight − present weight)/initial weight] × 100%.

### 4.3. Anthocyanin and Vitamin C (VC) Content

The anthocyanin content was determined as previously described by Teribia et al. [45], with slight modifications. About 5 g fruit was ground in liquid nitrogen and the sample was placed in 10 mL of acetone/acetic acid (99:1, *v*/*v*) solution. The absorbance values were measured at 510 nm and 700 nm using the pH differential method, and the anthocyanin content was calculated based on these values. Anthocyanin content was expressed as g kg^−1^. The VC content was determined as previously described by Galani et al. [46], with slight modifications. Briefly, 1 g of grape tissue was ground and mixed in 8 mL of precooled 5% metaphosphoric acid. The mixture was centrifuged, and the supernatant was collected. The reaction mixture comprised 400 μL of supernatant, 800 μL of phosphate-buffered saline (PBS), 800 μL of trichloroacetic acid, 800 μL of 44% phosphoric acid, 800 μL of 4% α, α′-bipyridine, and 400 μL of 3% ferric chloride. The absorbance of the solution was determined at 525 nm to calculate the VC content. VC content was expressed as g kg^−1^.

### 4.4. Malondialdehyde (MDA) and Hydrogen Peroxide (H_2_O_2_) Content

The MDA content was determined as previously described by Sun et al. [47], with some modifications. Briefly, 0.5 g of frozen grape tissue was ground and mixed with 5 mL of 10% trichloroacetic acid to form a homogenate. MDA content was determined using the Micro Malondialdehyde (MDA) Assay Kit (Elabscience, Wuhan, China), and the absorbance values of reactants were measured at 532 nm and 600 nm. The difference in the absorbance values at the two wavelengths was used to calculate the MDA content. The H_2_O_2_ content was determined as previously described by Ge et al. [48], with slight modifications. The absorbance of the supernatant was determined at 410 nm to measure the H_2_O_2_ content. Three replicates were used for each biological repeat in each experiment. MDA content was expressed as mmol kg^−1^.

### 4.5. Enzyme Activity Assays

Polygalacturonase (PG) and cellulase (Cx) activities were detected as previously described by Xue et al. [49]. Briefly, for PG activity measurement, 0.5 mL of the enzyme extraction solution was added to 1 mL of acetic acid–sodium buffer and 0.5 mL of polygalacturonic acid to obtained the reaction sample. In addition, 0.5 mL of boiled enzyme solution was used as control. The absorbance values of the solutions were detected at 540 nm. PG activity was expressed as the amount of galacturonic acid (µg) produced by the hydrolysis of polygalacturonic acid per hour. For Cx activity, 1.5 mL of 10 g L^−1^ carboxy methyl cellulose was added to 0.5 mL of the reaction sample, and the absorbance was measured at 540 nm. Again, 0.5 mL of boiled enzyme solution was used as a control. Cx activity was expressed as the amount of reducing sugar (µg) formed by the hydrolysis of cellulose per hour. Pectin esterase (PE) activity was determined, as previously described by Deng et al. [33,50], with some modifications. The PE enzyme activity test kit (Solarbio, Beijing, China) was used to determine PE activity. The volume consumed by NaOH was recorded to calculate PE activity. LOX activity was measured as previously described by Yu et al. [51], with some modifications. Briefly, 0.1 mL of the enzyme solution was mixed with 2.4 mL of 0.1 g/L linoleic acid, and the absorbance value of the solution was measured at 234 nm. Again, 0.5 mL of boiled enzyme solution was used as a control.

### 4.6. Transcriptome Deep Sequencing (RNA-seq) Library Preparation and Sequencing

Groups 1, 2, and 5 were analyzed using RNA-seq at 2, 4, and 6 d post-treatment. Briefly, total RNA was extracted from grapes, and its concentration and purity were detected using a NanoDrop 2000 (Thermo Scientific, Waltham, MA, USA) spectrophotometer. Oligo (dT) and poly (A) magnetic beads were used for A-T base pairing. mRNA was isolated from total RNA and further used to synthesize cDNA. Sequencing was performed on an Illumina NovaSeq 6000 platform, and the library was constructed using an Illumina Truseq™ RNA sample prep kit (Illumina, San Diego, CA, USA). RNA-seq was carried out at Majorbio Biopharm Technology Co., Ltd. (Shanghai, China).

The original sequencing data were filtered using SeqPrep and Sickle. High-quality clean data were obtained by removing reads with adaptor sequences, low-quality reads, reads with N (N represents uncertain base information), and short reads. The clean reads of each sample were compared with the specified reference genome. HISAT2 2.2.1 software was used for sequence alignment analysis (reference genome: https://urgi.versailles.inra.fr/Species/Vitis/Data-Sequences/Genome-sequences (accessed on 9 August 2019). Annotation information for genes was obtained from the NR and Swiss-Prot databases. The original data have been submitted to the NCBI database with the accession number PRJNA748249.

### 4.7. Analysis of DEGs

Gene expression was analyzed using the RSEM software (1.3.3) [52]. Differential expression of the genes was analyzed using DESeq2 [53]. The screening criteria for differential expression included |log_2_FC| > 1 and p-adjust < 0.05.

### 4.8. Gene Ontology (GO) and Kyoto Encyclopedia of Genes and Genomes (KEGG) Enrichment Analysis

Goatools 1.4.12 software and R scripts were used to analyze the enrichment of GO and KEGG pathways [54]. GO and KEGG pathways were considered significantly enriched when p-adjust < 0.05. In the DEG analysis, the results of the GO and KEGG analyses were mapped using the online Hiplot v0.1.7 software. For the weighted gene co-expression network analysis (WGCNA), the results of the GO and KEGG analyses were mapped using the online ImageGP v1.0 software.

### 4.9. Cluster Analysis and WGCNA

An R 4.4.1 software package was used for WGCNA [55]. Gene modules were identified, and the correlations between modules and treatments were determined. The connectivity degree of each gene was calculated to determine the hub genes in the model, and Gephi (0.9.2) was used to visualize the network.

### 4.10. Quantitative Real-Time Polymerase Chain Reaction (qRT-PCR)

Total RNA was extracted from grape samples using the Total RNA isolation Reagent Kit (Biosharp, Hefei, China). DNA was removed from the samples and reverse-transcribed to form cDNA using the PrimeScript™ RT reagent Kit (Takara, Dalian, China). Grape *ubiquitin1* was used as the internal reference gene. Data were analyzed using the 2^−ΔΔct^ method [56]. Three biological replicates were used for each treatment. The primers used for qRT-PCR are listed in Table S1.

### 4.11. Kinetic Parameters of Catalytic Reaction

The open reading frame (ORF) of *VlCOMT* was cloned into the *Sal*I/*Kpn*I site of the pMAL-c5X vector (New England Biolabs UK Ltd., Hitchin, UK). The fusion protein was purified using the pMAL system (New England Biolabs UK Ltd.). The Km refers to the concentration of S at half the Vmax of the enzymatic reaction. Its size is related to the nature of the enzyme. In general, the Lineweaver–Burk equation (double reciprocal plot method) is the most common method. In the present study, the substrate was configured with a certain concentration gradient solution, and the corresponding reaction rate, V, at each corresponding concentration, was determined. The measured 1/V vs. 1/[S] was plotted to obtain a straight line and the fitting equation: 1/V = Km/Vmax[S] + 1/Vmax.

### 4.12. High-Performance Liquid Chromatography (HPLC) Assay

The standards of caffeic acid, methyl caffeate, ferulic acid, and 4-hydroxy-3-methoxycinnamic acid methyl ester were dissolved in methanol/formic acid solution (95:5) and configured with certain concentrations to be analyzed as standards. A 10 mL reaction mixture was configured with 200 μM substrate, 1 mM SAM, 1 mM dithiothreitol, 200 mM Tris-HCl buffer solution (pH 7.0), and 10 μg/mL VlCOMT. After 25 min of incubation, the reaction was terminated by adding 1 mL of concentrated hydrochloric acid. After repeated extraction with the same volume of ethyl acetate thrice, the reaction solution was collected into a round-bottom flask and spun to dry. After solute precipitation, the methanol/formic acid solution (95:5) was added to it for dissolution. The solution was repeated three times. The washing liquid was collected and merged. The volume was set to 5 mL before being analyzed as a sample. The samples and standards were filtered with a 0.22 μm filter head for HPLC detection.

### 4.13. Determination of Lignin, Cellulose, and Hemicellulose Content

The samples were pretreated, crushed with a grinder, and dried at 85 °C. A combination of the 2 mol L^−1^ hydrochloric acid hydrolysis method and DNS method was used for hemicellulose quantification [57]. The potassium dichromate iodine method was used to determine cellulose levels. The lignin content of the samples was determined as previously described by Jung et al. [58]. Each experiment was conducted with three biological replicates.

### 4.14. Generation of Transgenic Tomato Plants

The complete coding sequence (CDS) of *VlCOMT* was amplified by PCR using the first-strand cDNA of tomato synthesized using a VlCOMT-specific primer. Subsequently, the *VlCOMT* CDS was inserted into the plant expression vector pSAK277. The resulting recombinant vector was then introduced into the tomato cultivar Micro-Tom using a tissue culture-based Agrobacterium-mediated stable transformation method (strain GV3101). RNA was extracted from young leaves and used for qRT-PCR to identify the positive transgenic lines. The specific primers used for qRT-PCR were 5′-GATATGAACTACACAATCCTCCTG-3′ (sense) and 5′-ATAAGTTATAACTCGGGTACGGAC-3′ (antisense). qRT-PCR was performed as described in Section 4.10.

### 4.15. Subcellular Localization of VlbZIP14

We PCR-amplified the ORFs for *VlbZIP14* and inserted the PCR products at the XbaI/BamHI restriction sites in the PBI211-GFP vector to form PBI211-GFP/VlbZIP14. Onion epidermis was transformed using the gene gun method [59]. Next, the transformed material was incubated in a hypertonic medium overnight at room temperature. The material was then incubated in an isotonic medium for 24 h. Finally, the green fluorescent protein (GFP) fluorescence was observed using a confocal laser scanning microscope (Olympus, Tokyo, Japan).

### 4.16. Dual Luciferase Assay

The full-length CDSs of transcription factors were cloned into separate pGreenII-62-SK vectors, and the *VlCOMT* promoter was cloned into the pGreenII-0800-LUC vector [37]. The recombinant plasmids were transformed into *Agrobacterium* GV3101 (pSoup). Then, the inducing fluid was infiltrated into *N. benthamiana* leaves. The tobacco plants were then grown for 2–3 days in the dark. Next, luminescence was measured using a live imaging apparatus. The fluorescence signal values were detected using the Infinite M200Pro Microplate System. Three biological replicates from each group were analyzed. To ensure experimental accuracy, firefly luciferase and Renilla luciferase luminescence were subtracted from those of blank control groups for each experimental group. Primers used for vector construction are listed in Table S2.

### 4.17. Yeast One-Hybrid (Y1H) Assay

The full-length *VlbZIP14* CDS was cloned and inserted into the pGADT7 vector. The G-box or a mutant G-box from the promoter regions of *VlCOMT* genes were separately inserted into the pABAi reporter vector and cultured on a selective medium (SD/-Leu/-Ura/+150 ng/mL AbA) for 2 d. For the *VlbZIP14* transactivation assay in yeast, the BD-VlbZIP14, BD-VlbZIP14Δ1, BD-VlbZIP14Δ2, BD-VlbZIP14Δ3, and BD-VvibZIP14Δ4 constructs were generated using the pGBKT7 backbone vector and the full-length or truncated forms of *VlbZIP14*. The two vectors were transformed into AH109 yeast-competent cells via the yeast rapid transformation method and were grown on SD/-Trp or SD/-Trp-Ade-His selective medium [60]. The cells were then incubated for 2–3 d at 30 °C, and their growth was monitored.

### 4.18. Statistical Analysis

Physiological data were analyzed using analysis of variance (ANOVA) in SPSS software (v21.0). Statistically significant differences in the qRT-PCR data were determined using Student’s *t*-tests with significance levels denoted as * *p* < 0.05, ** *p* < 0.01, and *** *p* < 0.001. Data were presented as mean ± SD (*n* = 3).

## 5. Conclusions

This study showed that SN treatment could maintain the storage quality of grapes postharvest and inhibit SO_2_-induced grape berry abscission. Transcriptome sequencing results showed that SN treatment affected the internal cell wall metabolism and other related pathways of grapes, with *VlCOMT* playing a central role as a key regulatory gene. Furthermore, *VlCOMT* is a direct target of *VlbZIP14*. Herein, we proposed a model for *VlCOMT* in response to the SN treatment (Figure 9). Our study provides a basis for further research to unravel the mechanism of action of *VlCOMT* in response to SN treatment to utilize it in the molecular breeding of table grapes to extend their shelf life.

## Data Availability

https://www.ncbi.nlm.nih.gov/bioproject/PRJNA748249/.

## Supplementary Materials

The following supporting information can be downloaded at: https://www.mdpi.com/article/10.3390/ijms25179479/s1.
