# Supplementary material for: Transcription Factor VlbZIP14 Inhibits Postharvest Grape Berry Abscission by Directly Activating VlCOMT and Promoting Lignin Biosynthesis
Source: Int J Mol Sci. 2024 Aug 31;25(17):9479. doi: 10.3390/ijms25179479 (PMC11395120; doi:10.3390/ijms25179479)
Supplement: Supplementary file 1 [file ijms-25-09479-s001.zip › ijms-3182691-supplementary.pdf]

Supplemental Figure

Fig. S1.

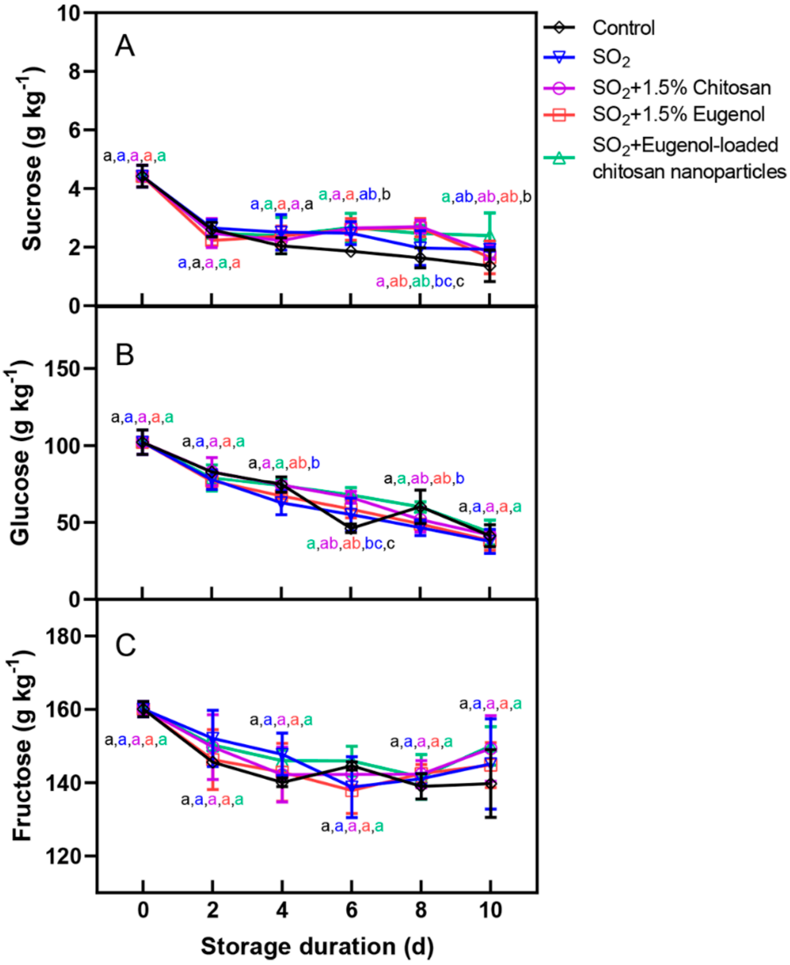

Fig. S1. Effect of exogenous treatments on fructose of table grape. (A) sucrose (B) glucose (C) fructose. The data represents the average ± SD（n=3）. Significant differences between means were determined using ANOVA ($P < 0.05$) and are denoted by different lowercase letters.

Fig. S2.

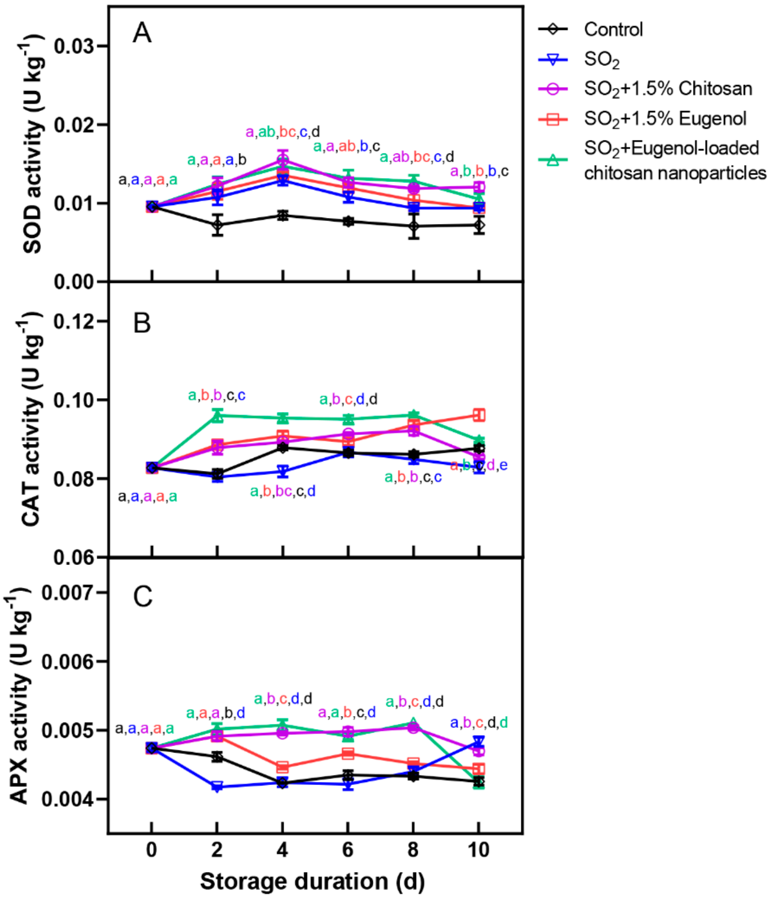

Fig. S2. Effect of exogenous treatments on the activity of (A) superoxide dismutase (SOD), (B) catalase (CAT), (C) ascorbate peroxidase (APX) of table grapes. Data represent average ± SD (n=3). Significant differences between means were determined using ANOVA ($P < 0.05$) and are denoted by different lowercase letters.

Fig. S3.

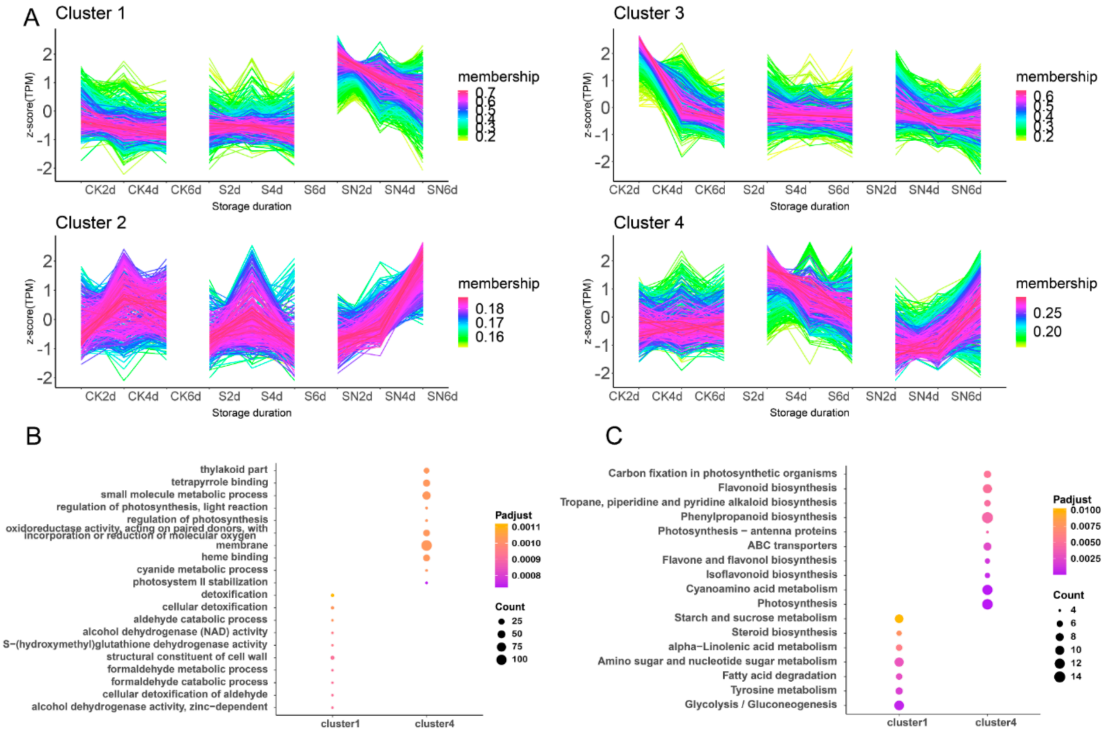

Fig. S3. Analysis of DEGs expression patterns during storage. (A) Cluster analysis of genes expression patterns after SO_2 and SN treatment during storage. S: SO_2 treatment; SN: SO_2+eugenol-loaded chitosan nanoparticles edible coating treatment. (B) GO enrichment analysis. (C) KEGG pathway analysis.

Fig. S4.

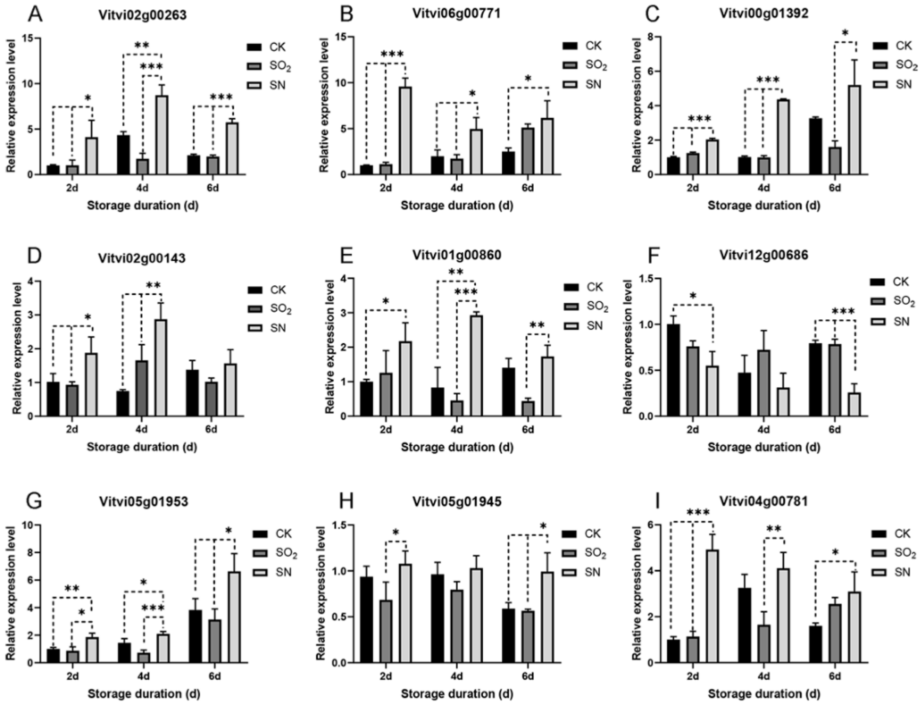

Fig. S4. Expression profiles of DEGs based on qRT-PCR. Three biological replicates were used for analysis. Asterisks indicate the level of significance (*P <0.05, **P < 0.01, and ***P < 0.001) according to Student's t-test.

Supplemental table

Table S1.

Primer sequences used for qRT-PCR analysis

Primer name	oligonucleotide primers	Purpose
Vitvi02g00263-F	TGCAGGGGATATGTTCACAAG	qRT-PCR
Vitvi02g00263-R	TGTCTGGTAATGCATGGTAGC	
Vitvi06g00771-F	ATCTGGAGCACATACAATAGGC	qRT-PCR
Vitvi06g00771-R	TGATCAGAGTGAAGAAGACCCT	
Vitvi00g01392-F	CAAACGGACGCCATACTGTC	qRT-PCR
Vitvi00g01392-R	CAACTGGGCACACTCAAGTC	
Vitvi02g00143-F	GACAACTCCTCCGAAATCTCC	qRT-PCR
Vitvi02g00143-R	CAACCCAGCAAAGTGAATGAC	
Vitvi01g00860-F	TGACTCAACTCGTCCGATCTA	qRT-PCR
Vitvi01g00860-R	AGCGTAGTTCAATGGGTTCTC	
Vitvi12g00686-F	AAGGAGGTGCAGATTGTAGC	qRT-PCR
Vitvi12g00686-R	AGCACCATTGAAGTAGCAGG	
Vitvi05g01953-F	AGTGCCGAAGACTCATACAAG	qRT-PCR
Vitvi05g01953-R	TAGGAGATGGTGGTTTGTGG	
Vitvi05g01945-F	AAGACTCATACAAGCCACCTC	qRT-PCR
Vitvi05g01945-R	ATGGGAGAAGGAGGTTTGAG	
Vitvi04g00781-F	TGTGGAAGCAGAAGTGGAAC	qRT-PCR
Vitvi04g00781-R	TAACCCAACATGATGCACCC	

Table S2.

Primers used for vector construction in this study

Primer name	Primer sequence(5'-3')	vector
Vitvi17g00809 (DOF5.6)-F	ACGATTCACACTCGGTAGCAG	Full length
Vitvi17g00809 (DOF5.6)-R	GACTAGTGGGTTTGTGGTTGATG	
Vitvi10g01594 (ERF4)-F	GTTGACTGTGCCCACCCTTTCTTAG	Full length
Vitvi10g01594 (ERF4)-R	TCCAGTGAGGACGCAAAGATCAGAG	
Vitvi06g00741 (WRKY24)-F	ATGGGTTCTTCCTCTGGGAGCTTAG	Full length
Vitvi06g00741 (WRKY24)-R	ACTCGAATAACATGTCGTCTCTGGG	
Vitvi05g00108 (bZIP14)-F	GATATGAACTACACAATCCTCCTG	Full length
Vitvi05g00108 (bZIP14)-R	ATAAGTTATAACTCGGGTACGGAC	
Vitvi15g01003 (WRKY53)-F	ATGGAAATGGCCAGGGAGTG	Full length
Vitvi15g01003 (WRKY53)-R	TCCAGCGGGAAACTGTAGTC	
Vitvi15g00839 (ANL2)-F	GAGACAGCAGACCAAGTACGAG	Full length

Vitvi15g00839 (ANL2)-R	TCAGCTTTCGCAGTGAAGGG	
Vitvi02g00757 (KAN2)-F	AGAGATTGGTGGAAGATGGAGTTG	Full length
Vitvi02g00757 (KAN2)-R	AGATATCATCAATGTGACCTCCCC	
Vitvi15g00912 (ATHB12)-F	GAGTAGTTGGTCTGAGGAGTTTTTG	Full length
Vitvi15g00912 (ATHB12)-R	CTTTGGACAGGGTAGTGAGATTAGC	
Vitvi14g00018 (MYB61)-F	CTGCCACTTCATTTTCTCAACCTC	Full length
Vitvi14g00018 (MYB61)-R	CCTTTTGATTGCTTACAGGGACAG	
Vitvi13g01266 (GAMYB)-F	ATGAGTCACTTGACAAATGAGAGC	Full length
Vitvi13g01266 (GAMYB)-R	AGGAAGTTCAGACATTTGACAGAC	
Vitvi08g01841 (NAC87)-F	CTCCTTCAATACAGGCTCTTCTCTC	Full length
Vitvi08g01841 (NAC87)-R	CAGGCTGCTTCTTCTTGTATCTTC	
Vitvi05g00715 (ERF92)-F	AGAGGAGCCACAAACAAATATCAC	Full length
Vitvi05g00715 (ERF92)-R	GATCAAGGACTAGAGGTCTCAGAG	
Vitvi02g00387 (HFB2B)-F	GGTTATCAGTTTCTTAGGGTTCGG	Full length
Vitvi02g00387 (HFB2B)-R	GCAAACCAACCTAACCTTGATGAG	
Vitvi11g00680 (bHLH66)-F	CTCAACCCTACCTCCCTCAAATG	Full length
Vitvi11g00680 (bHLH66)-R	AAATACCACATCAGACATCGGCA	
Vitvi10g01078 (WRKY14)-F	TCGGTATAACAGGTGCCAAACTC	Full length
Vitvi10g01078 (WRKY14)-R	CCTAGAGAGGCGAGATTGATAAG	
Vitvi10g00063 (WRKY6)-F	ATGGACAAAGGATGGGGTCTCAC	Full length
Vitvi10g00063 (WRKY6)-R	GTTGGTGTTGCTGGAGTTGGTTG	
Vitvi11g01188 (WRKY11)-F	ATGGCCGTCGATTTTCTAGG	Full length
Vitvi11g01188 (WRKY11)-R	GGTTGATGGGAACATGAGCC	
Vitvi10g00854 (ARF6)-F	ATGAGGTTGTCTCCTGCTGG	Full length
Vitvi10g00854 (ARF6)-R	GTAGTCAAGAGACCCCACAG	
Vitvi06g00360 (RAP2-7)-F	GATGTTTTCTCTCCGACTCCGAC	Full length
Vitvi06g00360 (RAP2-7)-R	GGAGGTGTGATGGGTGGATTAAG	
Vitvi05g01733 (MYB15)-F	GCCAGAGGTTAGAGTAGTGGTGTTC	Full length
Vitvi05g01733 (MYB15)-R	ATGCATCACTATGTTCAGATCCTCC	

Vitvi10g00618 (WRKY65)-F	CGTCCTTCATAAACTATTCCTCTG	Full length
Vitvi10g00618 (WRKY65)-R	ATCTCAGCTTGTGGTCCCACAC	
Vitvi08g01336 (DIV)-F	GTCTCTGTACTCATTCCTAGCACTG	Full length
Vitvi08g01336 (DIV)-R	CTTATCTCACTAGCGCCTCTATTTC	
